# Evolution of the Araliaceae family inferred from complete chloroplast genomes and 45S nrDNAs of 10 *Panax*-related species

**DOI:** 10.1038/s41598-017-05218-y

**Published:** 2017-07-07

**Authors:** Kyunghee Kim, Van Binh Nguyen, Jingzhou Dong, Ying Wang, Jee Young Park, Sang-Choon Lee, Tae-Jin Yang

**Affiliations:** 1 Department of Plant Science, Plant Genomics and Breeding Institute, and Research Institute of Agriculture and Life Sciences, College of Agriculture and Life Sciences, Seoul National University, Seoul, 151-921 Republic of Korea; 2 School of Forestry and Horticulture, Hubei University for Nationalities, Enshi, 445000 China; 3 Key Laboratory of South China Agricultural Plant Molecular Analysis and Genetic Improvement, Provincial Key Laboratory of Applied Botany, South China Botanical Garden, Chinese Academy of Sciences, Guangzhou, Guangdong Province 510650 China; 4 Crop Biotechnology Institute/GreenBio Science and Technology, Seoul National University, Pyeongchang, 232-916 Republic of Korea

## Abstract

We produced complete sequences and conducted comparative analysis of the maternally inherited chloroplast (cp) genomes and bi-parentally inherited 45S nuclear ribosomal RNA genes (nrDNA) from ten Araliaceae species to elucidate the genetic diversity and evolution in that family. The cp genomes ranged from 155,993 bp to 156,730 bp with 97.1–99.6% similarity. Complete 45S nrDNA units were about 11 kb including a 5.8-kb 45S cistron. Among 79 cp protein-coding genes, 74 showed nucleotide variations among ten species, of which *infA, rpl22*, *rps19* and *ndhE* genes showed the highest Ks values and *atpF*, *atpE, ycf2* and *rps15* genes showed the highest Ka/Ks values. Four genes, *petN*, *psaJ*, *psbF*, and *psbN*, related to photosynthesis and one gene, *rpl23*, related to the ribosomal large subunit remain conserved in all 10 Araliaceae species. Phylogenetic analysis revealed that the ten species could be resolved into two monophyletic lineages, the *Panax-Aralia* and the *Eleutherococcus-Dendropanax* groups, which diverged approximately 8.81–10.59 million years ago (MYA). The *Panax* genus divided into two groups, with diploid species including *P. notoginseng*, *P. vietnamensis*, and *P. japonicus* surviving in Southern Asia and a tetraploid group including *P. ginseng* and *P. quinquefolius* Northern Asia and North America 2.89–3.20 MYA.

## Introduction

The Araliaceae (also known as the ginseng family) and the Apiaceae are the major families in the order Apiales belonging to Asterid II^1–3^. The Araliaceae family comprises 55 genera and more than 1,500 plant species widely distributed in tropical, subtropical and temperate regions^4, 5^, many of which are used as oriental medicines, such as species in the genus *Panax*, *Eleutherococcus* and *Aralia* ^6, 7^. According to taxonomical studies, Araliaceae encompasses two large monophyletic groups: the *Aralia-Panax* group and the Asian Palmate group^8^. The *Aralia-Panax* group consists of the two closely-related genera, *Aralia* and *Panax*. Meanwhile, the Asian Palmate group is represented by the genera *Eleutherococcus*, *Dendropanax*, and *Schefflera* characterized as distinctive woody plants.

Although the conserved basic chromosome number was estimated to be x = 12 in Araliaceae family species based on diploid taxa (2n = 24), the chromosome numbers vary from 2n = 48 to 2n = 192 in polyploid species of the family^9, 10^. In the genus *Panax*, *P. notoginseng*, *P. vietnamensis* and *P. japonicus* are diploid with chromosome number of 2n = 24, while *P. ginseng* and *P. quinquefolius* are considered to be tetraploid with chromosome numbers of 2n = 48. The genus *Aralia* and *Eleutherococcus* are reported to have various chromosome numbers including 2n = 24, 36 or 48. (CCDB-chromosome count database; http://ccdb.tau.ac.il/).

Although many studies have reported taxonomical classification and divergence of Araliaceae species based on molecular data derived from a few chloroplast (cp) and nuclear sequences^11–16^, genetic diversity surveys and molecular phylogenetic classification of *Panax* and its relatives are still very limited. Cytoplasmic cp genomes and nuclear ribosomal DNA have widely been used to elucidate the evolution of plant species, owing to their characteristic highly conserved sequences, such that minor sequence divergences reflect evolutionary history^17–20^. Recently, we developed a *de novo* assembly method to obtain complete cp and nrDNA sequences using low-coverage whole-genome sequence (dnaLCW) and applied it to reveal the evolutionary history of various plant lineages such as *Oryza* AA genomes and *Epimedium* species^21, 22^, and also to identify intra-species diversity^23^.

In this study, we characterized cp genomes and 45S nrDNA sequences of ten Araliaceae species including *Panax*, *Aralia*, *Eleutherococcus*, and *Dendropanax* species and investigated genetic diversity among them to understand diversity and molecular evolution of the Araliaceae species.

## Results

### Complete chloroplast genomes and 45S nrDNA sequences

Novel complete cp genomes of five species, *P. notoginseng*, *P. japonicus*, *P. vietnamensis*, *A. elata*, and *E. sessiliflorus*, and 45S nrDNA sequences of seven species, *P. quinquefolius*, *P. notoginseng*, *P. japonicus*, *P. vietnamensis*, *A. elata*, and *E. sesiliflorus*, and *D. morbifera*, were characterized in this study (Table 1).

**Table 1:** Cp genomes and 45S nrDNA sequences used for comparative analysis in this study.

Genus	Species (Abbreviated name)	WGS reads used			Length (GenBank accession no.)	
		Amounts (Mb)	Cp Coverage (x)	45S nrDNA Coverage (x)	Cp	45S nrDNA (bp)
*Panax*	*P. ginseng* (PG)	505	97	659	156,248 (KM088019)	11,091 (KM036295)^b^
	*P. quinquefolius* (PQ)	1,010	127	533	156,088 (KM088018)	11,169 (KM036297)^c^
	*P. notoginseng* (PN)	2,811	246	2555	156,466 (KP036468)	6,306 (KT380921)^b^
	*P. japonicus* (PJ)	2,870	237	4295	156,188 (KP036469)	6,275 (KT380920)^b^
	*P. vietnamensis* (PV)	4,586	1,005	2267	155,993 (KP036470)	7,280 (KT380922)^b^
*Aralia*	*A. elata* (AE)	505	90	267	156,220 (KT153023)	6,073 (KT380919)^b^
	*A. undulata* (AU)	NA	NA	NA	156,333 (NC_022810)^a^	610 (AF273540)^d^
*Eleutherococcus*	*E. sessiliflorus* (ES)	468	57	426	156,730 (KT153019)	10,109 (KT380924)^c^
	*E. senticosus* (ESen)	NA	NA	NA	156,768 (NC_016430)^a^	610 (AB570259.1)^d^
*Dendropanax*	*D. morbifera* (DM)	3,453	222	1124	156,366 (KR136270)	9,332 (KT380923)^b^

^a^Cp sequences retrieved from GenBank. ^b^45S nrDNA including full 45S transcription sequence (5.8 kb) and partial IGS sequence. ^c^45S nrDNA including full 45S transcription sequence (5.8 kb) and full IGS sequence. ^d^nrITS (ITS1-5.8S-ITS2) sequences retrieved from GenBank. NA: not available.

Complete lengths of the cp genomes ranged between 155,993 bp (*P. vietnamensis*) and 156,730 bp (*E. sessiliflorus*), with average read-mapping coverages of 57× to 1,005× (Table 1). The 45S nrDNA sequences were assembled into single contig for each of the seven species. The sequence lengths of the complete 45S nrDNA unit was around 11 kb including full intergenic spacer (IGS) sequences (Table 1). The 45S nrDNA sequences of *P. quinquefolius* and *E. sessiliflorus* were full-length sequences covering a 45S transcription unit (18S-ITS1–5.8S-ITS2-26S) and complete IGS sequences, whereas those of the other five species (*P. notoginseng*, *P. japonicus*, *P. vietnamensis*, *A. elata* and *D. morbifera*) consisted of 45S nrDNA transcription regions and partial IGS sequences (Table 1).

### Diversity of cp genome sequences

**Figure 1:**
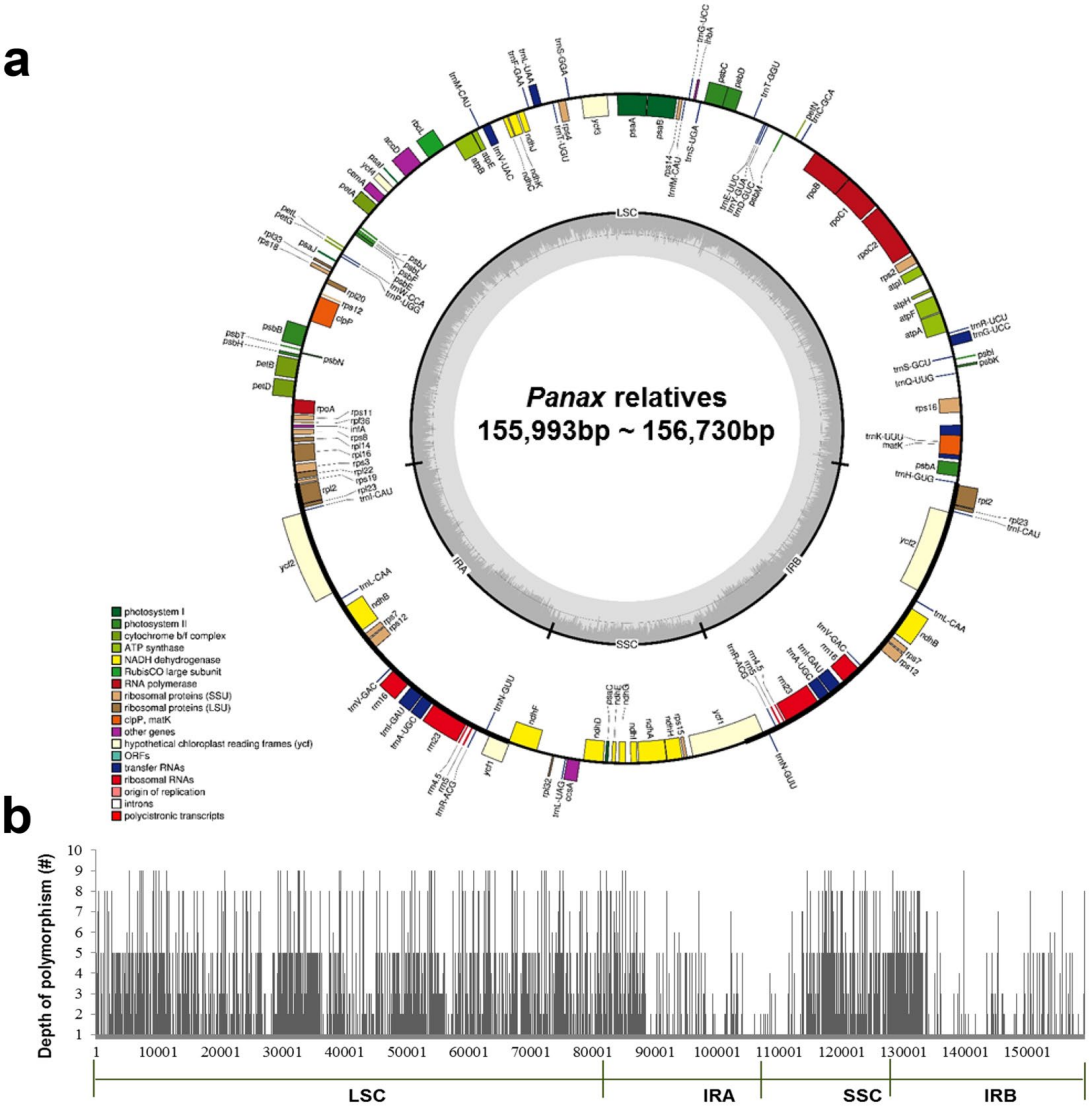
Gene map and nucleotide polymorphism of cp genomes in ten Araliaceae species. (**a**) Colored boxes are conserved chloroplast genes classified based on product function. The complete cp genome sequence was generated by the dnaLCW method and annotated using the DOGMA program (http://dogma.ccbb.utexas.edu/). The map was prepared using OGDRAW (http://ogdraw.mpimp-golm.mpg.de/). Genes transcribed clockwise and counterclockwise are indicated on the outside and inside of the large circle, respectively. (**b**) The depth of polymorphisms found among cp genomes of ten Araliaceae species. Cp genome (KM088019) of *P. ginseng* was used as a reference for comparison.

In addition to the five cp genomes described above, we used cp genomes of three species, *P. ginseng*, *P. quinquefolius*, and *D. morbifera* that were obtained in our previous study^23–25^. Cp genome sequences for two Araliaceae species, *A. undulata*, *E. senticosus*, were also retrieved from GenBank for comparative analysis (Table 1). The gene order and gene content were highly conserved among the ten cp genomes (Fig. 1a), and the complete cp genome sequences showed 97.1~99.6% similarity among ten species (Supplementary Table S1). With regard to the quadripartite structure of the cp genome, nucleotide polymorphisms were lower in the inverted repeat regions (IRs) than in large single copy (LSC) and small single copy (SSC) regions (Fig. 1b). The cp genomes of five *Panax* species shared more than 98.9% similarity, among which *P. ginseng* and *P. quinquefolius* showed the highest similarity over 99.6% at both whole cp genome and cp coding sequence levels, respectively (Supplementary Table S1).

**Figure 2:**
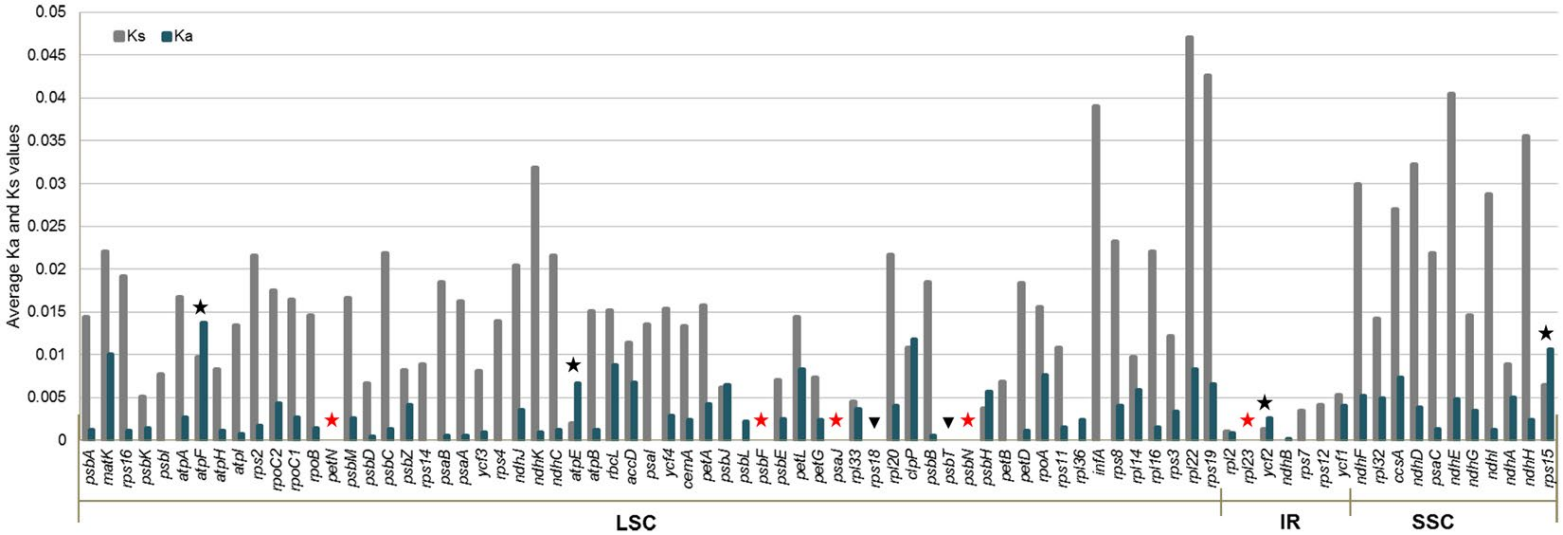
Summary of Ks and Ka values among cp genomes of ten species. The rates of non-synonymous substitution (Ka) and synonymous substitution (Ks) of 79 conserved protein-coding sequences were calculated and averaged using KaKs calculator. The average Ks and Ka values are indicated by grey and dark blue bars, respectively. Black stars indicate the genes evolved under positive selection pressure with over 1 for the value of Ka/Ks ratios. Red stars indicate five conserved genes without variations and black triangles indicate two conserved genes without SNP but with 21 and 6 bp InDel variations in Araliaceae family.

The sequence polymorphism rates of conserved coding regions were from 0.4~1.8% among ten Araliaceae species, in which the rate between *P. ginseng* and *P. quinquefolius* was the lowest (0.4%). Five genes, namely *petN*, *psaJ*, *psbF*, *psbN*, and *rpl23*, showed no sequence polymorphism among the ten Araliaceae species (Fig. 2 and Supplementary Dataset S1, Supplementary Fig. S4). The *ndhF* genes in cp genomes of *Aralia*, *Eleutherococcus* and *Dendropanax* species were 97 bp, 36 bp and 18 bp farther from the border junction of IRa and SSC, compared to those in cp genomes of *Panax* species (Supplementary Fig. S1).

The average Ks values for all protein-coding genes except cp-rRNA genes were from 0.0014 to 0.0199 and 0.0014 to 0.0068 at the inter- and intra-genus levels, respectively. The lowest Ks value of 0.0014 was between the two closest *Panax* species, *P. ginseng* and *P. quinquefolius*. The average Ks value among five species within the *Panax* genus was lower (0.0056) than those between *Aralia-Panax* (0.0110) and *Eleutherococcus-Dendropanax* (0.0080) (Table 2). The highest Ks values were detected in *infA, rpl22*, *rps19* and *ndhE* genes, while significantly high Ka/Ks ratios of more than 1 were detected in *atpF*, *atpE, ycf2* and *rps15* genes (P-value 8.3E-27–3.2E-2, Chi-square test) (Fig. 2).

**Table 2:** Ks values and estimated divergence time of 10 Araliaceae species.

Species		Divergence time (MYA)^b^									
		PG	PQ	PJ	PV	PN	AU	AE	Esen	ES	DM
**Average Ks** ^**a**^	**PG**		0.72	3.41	3.30	3.05	8.41	8.28	8.70	9.37	8.27
	**PQ**	0.0014		3.30	3.18	2.93	8.28	8.15	8.57	9.32	8.06
	**PJ**	0.0068	0.0066		1.69	3.15	8.85	8.72	9.20	9.95	8.72
	**PV**	0.0066	0.0064	0.0034		3.01	8.62	8.53	8.98	9.74	8.48
	**PN**	0.0061	0.0059	0.0063	0.0060		8.46	8.26	8.82	9.58	8.38
	**AU**	0.0168	0.0166	0.0177	0.0172	0.0169		3.20	8.33	9.13	8.11
	**AE**	0.0166	0.0163	0.0174	0.0171	0.0165	0.0064		8.30	9.09	7.96
	**ESen**	0.0174	0.0171	0.0184	0.0180	0.0176	0.0167	0.0166		2.98	4.10
	**ES**	0.0187	0.0186	0.0199	0.0195	0.0192	0.0183	0.0182	0.0060		4.85
	**DM**	0.0165	0.0161	0.0174	0.0170	0.0168	0.0162	0.0159	0.0082	0.0097	

^a^Average Ks values between common cp protein coding genes of each species calculated using KaKs calculator program. ^b^Divergence time was estimated by Ks/2λ, where λ = 1.0 × 10^−9^. PG to DM indicate abbreviated species name (Table 1).

**Figure 3:**
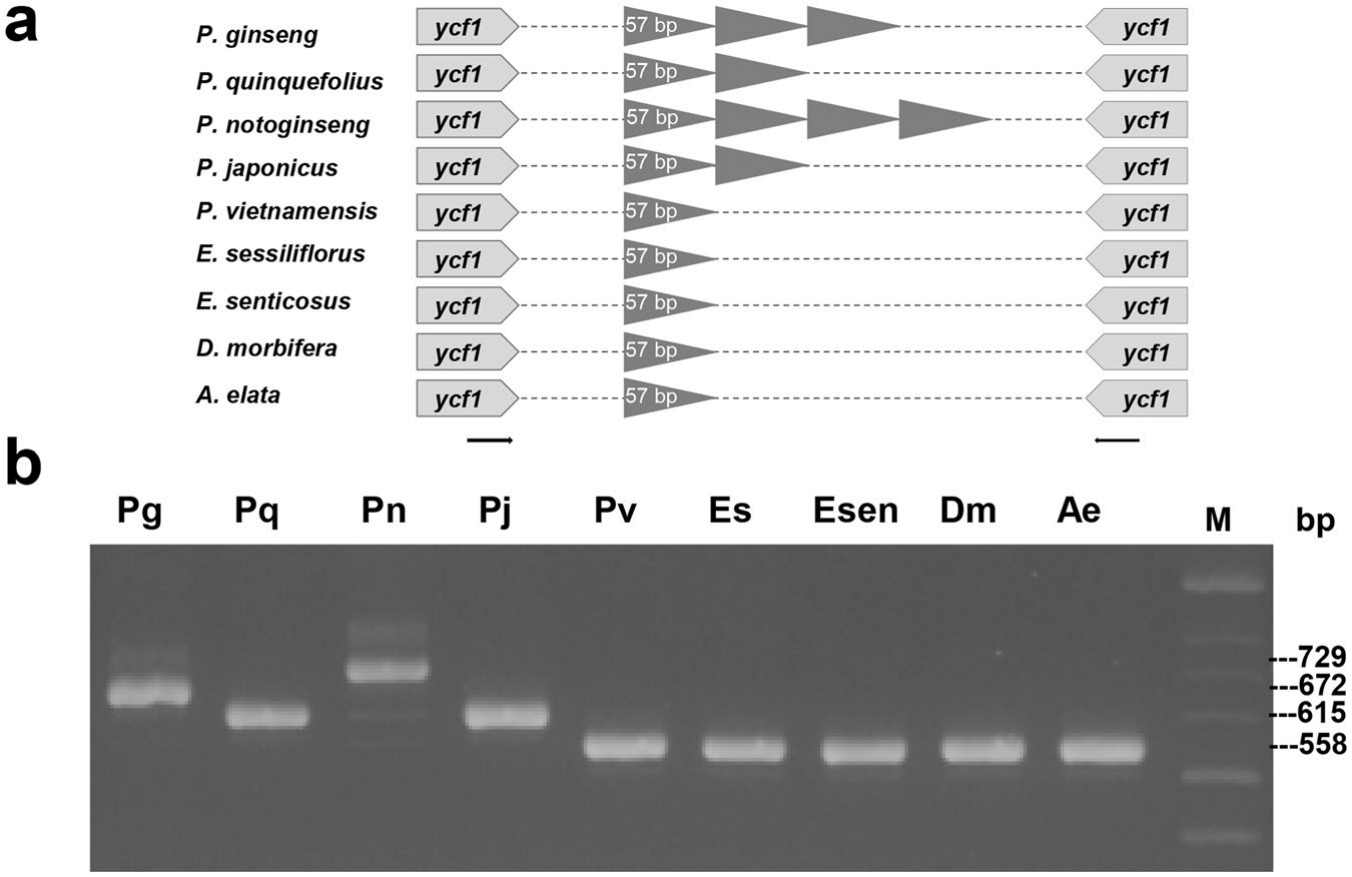
Validation of polymorphic sites with TR CNV in cp genomes of Araliaceae species. (**a**) Schematic diagram of CNV of TR units found in *ycf1* genes among nine species, and triangles indicate TR unit of 57 bp. (**b**) Validation of CNV of 57-bp TR in nine species using genomic DNA PCR analysis with the pgycf01 primer set (Supplementary Table S2). Abbreviated species names (Table 1) are shown above the lanes. M indicates 100-bp DNA ladder.

Although repeat motifs were diverse among cp genomes, conserved common repeat motifs were also present at both the intra-genus and inter-genus levels. Tandem repeat (TR) units with sizes of 6 to 12 bp were abundant among ten cp genomes (Supplementary Fig. S2). High copy number variation (CNV) for TRs was detected in the *ycf1* protein-coding gene and in the intergenic regions between *trnC*-GCA and *petN*, *trnS-*GGA and *rps4*, *trnT-*UGU and *trnL-*UAA, and *cemA* and *petA* (Fig. 3 and Supplementary Table S2, Supplementary Fig. S3).

### Sequence variations of nrDNA sequences in *Panax* and relatives

**Figure 4:**
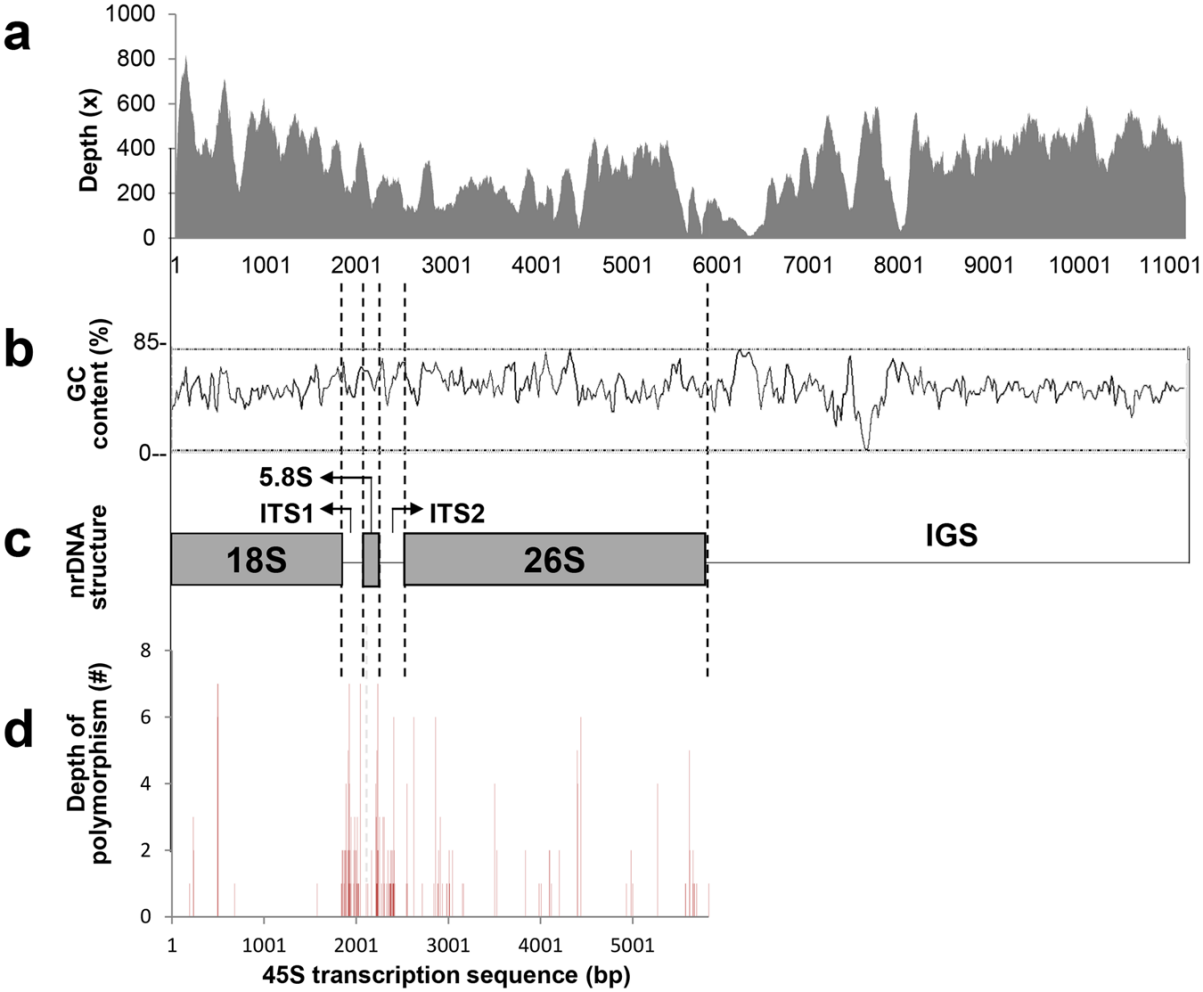
Assembly and comparison of 45S nrDNA sequences. (**a**–**c**) Full-length 45S nrDNA sequence of *P. quinquefolius* assembled in this study. Average read depth was 376× (**a**) and average GC content was 53.15% (**b**). (**c**) Schematic diagram of 45S nrDNA structure. (**d**) Comparison of 45S nrDNA transcription sequences of eight Araliaceae species. *P. ginseng* 45S nrDNA transcription sequence (KM036295) was used as reference for sequence comparison.

In addition to seven 45S nrDNA sequences assembled in this study, 45S nrDNA sequence of *P. ginseng* and nrITS sequences of *A. undulata* and *E. senticosus* were retrieved from GenBank and used for comparative analysis (Table 1). When 45S nrDNA transcription sequences (18S-ITS1-5.8S-ITS2) were compared among eight species (excluding *A. undulata* and *E. senticosus* for which there was no available sequence), sequence polymorphisms were found in genic regions as well as in two ITS regions although the polymorphisms were more relatively frequent in two ITS regions (Fig. 4 and Supplementary Tables S3 and S4); 9, 4, 50, 42, and 44 SNPs in 18S (1,808 bp), 5.8S (160 bp), 26S (3,452 bp), ITS1 (223 bp) and ITS2 (233 bp) regions, respectively. The numbers of SNPs in the 45S nrDNA sequence were higher at the inter-genus level than at the intra-genus level (Supplementary Tables S3 and S4).

### Phylogenetic analysis and divergence time estimation

**Figure 5:**
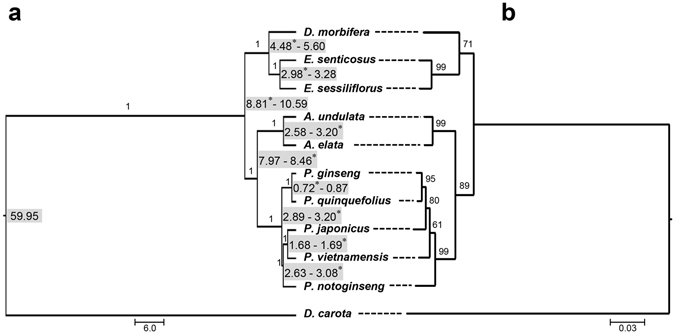
Phylogenetic tree and divergence time of 10 Araliaceae species. (**a**,**b**) Phylogenetic trees were generated based on cp protein-coding sequences (**a**) and nrDNA ITS sequences (ITS1-5.8S-ITS2) (**b**). Dashed lines connect the positions of each species in the two trees. Numbers next to nodes indicate average divergence time (*) based on Ks values (Table 2), and median divergence time using the BEAST program with 95% highest posterior density. The number above each branch refers to the Bayesian posterior probability (**a**) and ML bootstrap values (**b**).

A phylogenetic tree based on cp protein coding sequences showed two typical monophyletic lineages consisting of the *Aralia-Panax* group and the *Eleutherococcus-Dendropanax* group, which was also confirmed by a phylogenetic tree based on nrITS sequences (Fig. 5). Species of each genus, *Panax*, *Aralia*, *Eleutherococcus*, and *Dendropanax*, were grouped separately. The five *Panax* species were divided into two subgroups, in which one included *P. ginseng* and *P. quinquefolius* and the other included the remaining three *Panax* species (Fig. 5a). The topology of the tree based on nrITS sequences was almost identical to that in the cp sequence-derived tree, except for some differences among the *Panax* species (Fig. 5b).

Based on cp protein coding sequences, the divergence time between the *Aralia-Panax* group and the *Eleutherococcus-Dendropanax* group could be estimated at approximately 8.81–10.59 MYA (Fig. 5a). In the *Eleutherococcus-Dendropanax* clade, *Dendropanax* and *Eleutherococcus* were estimated to have diverged 4.48–5.60 MYA. In the *Aralia-Panax* clade, *Aralia* and *Panax* were estimated to have diverged 7.97–8.46 MYA, with subsequent speciation within these two genera likely to have occurred during a period approximately 2.89–3.20 and 2.58–3.20 MYA in *Panax* and *Aralia*, respectively. The two closest *Panax* species, *P. ginseng* and *P. quinquefolius*, were predicted to have diverged approximately 0.72–0.87 MYA.

## Discussion

### Genetic diversity in cp genomes of the Araliaceae family

Sequence variation was low throughout cp protein coding sequences and intergenic sequences among the ten Araliaceae species examined herein, although the cp protein coding sequences were more conserved (0.4~1.8% polymorphism) compared to intergenic sequences (0.4~2.9% polymorphism). Based on Ks values and divergence time inferred in this study, the substitution rate among cp genomes of *Panax* and relatives was approximately 1.0 × 10^−9^ per year, which seems to be consistent with a previous report^26^.

Based on analysis of non-synonymous and synonymous SNPs, average Ks and Ka values in LSC, SSC and IR regions among *Panax* relatives were low and ranged from 0.0022 to 0.0236, and from 0.0011 to 0.0046, respectively. Overall, genes in IR regions showed lower Ks values than did those in LSC and SSC regions. The ratio of the two parameters, Ka/Ks, is defined as the degree of evolutionary change in plants^27^. Among the protein-coding genes in the ten Araliaceae cp genomes, *atpF*, *atpE, ycf2*, and *rps15* were identified as being under positive selection (Fig. 2). Some of these genes were also reported to be related to evolution under positive selection pressure in Orobanchaceae and *Sesamun indicum* ^28, 29^. Among these five genes, *atpF* is a group II intron-containing gene with a known mechanism of splicing and heterogeneity in intron sequence^30, 31^.

### Five cp genes conserved in the Araliaceae family

We identified five identical genes which showed no sequence variation among all 10 Araliaceae species. Among the five conserved genes, four (*petN*, *psaJ*, *psbF*, and *psbN*) were related to photosynthesis and one (*rpl23*) was related to the ribosomal large subunit (Fig. 2 and Supplementary Dataset S1). We compared the five genes with those of *Daucus carota* belonging to the sister family Apiaceae in the Apiales order and to those of *Arabidopsis thaliana* in the Brassicales order (Supplementary Fig. S4). The *psbF* gene in the Araliaceae species was identical to that of *D. carota* but differed from that of *A. thaliana*, suggesting that the gene may be highly conserved in the Apiales order. Four of the five conserved genes showed sequence variation with orthologs of *D. carota*, indicating that these four genes are specifically conserved only in the Araliaceae species (Supplementary Fig. S4). It will be interesting to further elucidate the role of these five genes in the evolution of Araliaceae family.

### Nucleotide variation in ribosomal genes

Comparative analysis of eight 45S nrDNA sequences revealed that nucleotide polymorphism was much higher in nrITS regions than in transcribed ribosomal genes, 18S, 5.8S and 26S, as observed in other species^32, 33^. Most genetic diversity studies in plants have focused on ITS1 and ITS2 regions^34–38^. However, our study demonstrates that there is sufficient genetic diversity in the ribosomal gene regions for evolutionary analysis (Fig. 4 and Supplementary Tables S3 and S4). Among ribosomal genes, 26S was more divergent than 18S and 5.8S among the 10 Araliaceae species. Consistent with this finding, the 26S rDNA sequences have been applied for phylogenetic and divergence studies in yeasts^39^. The inter-genus level polymorphism was approximately three times higher than the intra-genus polymorphism (Supplementary Tables S3 and S4). Overall, our analysis suggests that 26S ribosomal gene sequences, in addition to the widely used ITS regions, can also be good targets for genetic diversity analyses for broad range and over genus-level taxon identification in the plant kingdom.

### Phylogeny based on cpDNA and nrDNA of Araliaceae

Two monophyletic groups, *Aralia-Panax* and *Eleutherococcus-Dendropanax*, were simultaneously confirmed with both cpDNA-based and nrITS-based trees. The finding that *Aralia* and *Panax* were the most closely related genus is in accordance with previous reports^15^. The *Panax* genus was clearly classified as two groups, with diploid species spread in Southern Asia including *P. notoginseng*, *P. vietnamensis*, *and P. japonicas* and a tetraploid group widely distributed in Northern Asia and North America including *P. ginseng* and *P. quinquifolius* (Fig. 5a).

The cpDNA-derived topology reflects the uniparental inheritance of cpDNA and agrees with the previously proposed models for evolution of these five *Panax* species. However, our nrITS-derived tree did not clearly distinguish between tetraploid and diploid groups (Fig. 5b). The different topology for the *Panax* genus between the cpDNA-based and nrITS-based trees may be caused by differences inherent to cpDNA and nrDNA in hybrid species including polyploids. According to previous reports, nrDNA (or nrITS)-based trees occasionally lack clear resolution in hybrid species (included polyploids) or closely related groups^40^ owing to low substitution rates in nrDNA. The nrDNA homogenized by inter-locus concerted evolution can give rise to multiple-heterogeneous types, especially in hybrid speciation. Under homogenization conditions, analysis based solely on nrDNA (or nrITS) sequence can produce misleading phylogenetic results in closely related species and polyploids (e.g. allopolyploids). Therefore, we recently suggested utilization of both cpDNA-based and nrDNA-based trees as a method conducive to determining well-resolved taxonomical positions of inter-subspecies hybrids among *Oryza* AA genomes^22^.

### Molecular clocks for speciation of *Panax* relatives

Based on previous reports, Araliaceae and Apiaceae diverged from a common ancestor in the Apiales order approximately 60.2 MYA^41–44^. In this study, we demonstrated that the Araliaceae family diverged into two monophyletic lineages approximately 8.81–10.59 MYA, followed by divergence of genera. *Aralia* and *Panax*, the two closest-related genera, diverged around 7.97–8.46 MYA, and *Dendropanax* and *Eleutherococcus* diverged after that (4.48–5.60 MYA).

In the *Panax* genus, we inferred speciation to have occurred more recently, around 2.89–3.20 MYA. This speciation step seems, like other plant speciation events, to be related to an Asian temperate climate change at that time period corresponding to uplift of the Himalaya-Tibetan plateau (in the late Cenozoic era)^45–47^. The Himalayas and southwestern China were suggested to be the base of speciation of *Panax* ^8^, and also reported to have a high rate of polyploidy because of the appearance of diverse species in the widespread alpine environment in these regions^48^. Earlier studies showed that *P. ginseng* underwent a recent whole-genome duplication 2.3 MYA^49^, followed by divergence of *P. ginseng* and *P. quinquefolius* ^50^. Our molecular clock estimation using complete cp coding genes supports the previous estimate and provides stronger evidence for that evolution scenario. *P. ginseng* might have evolved from allotetraploidization of diploid ancestors triggered by a large Asian temperate climate change. The diploid ancestors would have been isolated to the Northern hemisphere by uplift of the Himalaya-Tibetan plateau 2.89–3.20 MYA. We estimated divergence time between *P. ginseng* and *P. quinquefolius* to be approximately 0.72–0.87 MYA, i.e., following the recent genome duplication event at 2.3 MYA^49^. *P. quinquefolius* was suggested to have settled in North America by migration of the *P. ginseng* seeds via glacial movement of the Bering land bridge from eastern Asia to northern America and disjunction by geographical isolation 0.9–2.3 MYA^51^.

## Conclusion

The complete sequences of cp genomes and 45S nrDNA obtained in this study will contribute toward further understanding of evolution in the Apiales order. Phylogenetic and evolutionary analysis of cp genomes indicated that the evolutionary divergence of Araliaceae family produced two monophyletic lineages, followed by diversification of genera and speciation. Our findings demonstrated that simultaneous utilization of cp genomes and nrDNAs support fine-scale resolution of the evolutionary and taxonomical relationships of *Panax* and relatives in the Araliaceae.

## Materials and Methods

### Plant materials

Plant samples of five *Panax* species (*P. ginseng*, *P. quinquefolius*, *P. notoginseng*, *P. vietnamensis* and *P. japonicus*) and four relative species (*Aralia elata*, *Eleutherococcus sessiliflorus*, *E. senticosus* and *Dendropanax morbifera*) were used in this study (Table 1). Leaves were harvested from *P. ginseng*, *P. quinquefolius*, and *D. morbifera* plants grown in a ginseng research field (Seoul National University, Suwon, Korea), and from *P. vietnamensis* wild plants collected from Dak To district, Kon Tum province, Vietnam. Roots of *P. notoginseng* and *P. japonicus* were collected from Dafang County, Guizhou province and Enshi County, Hubei province, China, respectively. Leaves of *A. elata*, *E. sessiliflorus* and *E. senticosus* were from the farm of the Susinogapy Corporation (Cheonan, Korea, www.susinogapy.com).

### Genomic DNA isolation and whole-genome shotgun sequencing

Total genomic DNAs were isolated from tissue samples of the nine species using a modified cetyltrimethylammonium bromide (CTAB) method^52^ and examined using a spectrometer and agarose-gel electrophoresis. Illumina paired-end (PE) libraries were constructed with 300-bp insert size for each of eight species and sequenced using MiSeq or NextSeq platform by LabGenomics (Seongnam, Korea, http://www.labgenomics.co.kr/). Sequences of five species, *P. notoginseng*, *P. vietnamensis*, *P. japonicus*, *A. elata*, and *E. sessiliflorus*, were newly obtained in this study.

### *De novo* assembly and annotation of cp genomes and 45S nrDNA sequences

Complete cp genomes and 45S nrDNA sequences were assembled by *de novo* assembly with the low-coverage whole-genome sequence (dnaLCW) method^22^. Assembly errors and gaps were manually corrected by mapping of raw PE reads^22^

Structural features and genes in cp genomes were predicted using the DOGMA program (http://dogma.ccbb.utexas.edu/) and manual curation based on BLAST searches. Circular maps of cp genomes were made using OGDRAW (http://ogdraw.mpimp-golm.mpg.de/). The structures of 45S nrDNA sequences were predicted by comparison with reported *P. ginseng* 45S nrDNA sequence (KM036295) and analyses using RNAmmer (http://www.cbs.dtu.dk/services/RNAmmer/), and BLAST searches.

### Comparative analysis of cp genomes and 45S nrDNA sequences

Among the ten species, complete cp genomes of two species, *A. undulata* ^12^ (NC_022810) and *E. senticosus* ^53^ (NC_016430.1) were retrieved from GenBank. In addition, ITS1-5.8S-ITS2 (hereafter, nrITS) sequences of two species, *A. undulata* (AF273540) and *E. senticosus* (AB570259) were also retrieved from GenBank. To identify inter-species polymorphism, complete cp genome sequences of ten species were aligned and compared using MAFFT (http://mafft.cbrc.jp/alignment/server/) and mVISTA (http://genome.lbl.gov/vista/mvista/submit.shtml). For 45S nrDNA comparison, nrDNA sequences were extracted and compared among eight species, as for cp genomes. Tandem repeats (TRs) present on cp genomes of ten species were investigated using Tandem Repeat Finder (https://tandem.bu.edu/trf/trf.html).

### Validation of polymorphic sites

Among polymorphic sites found in cp genomes, those with copy number variation (CNV) of TRs and InDels were selected for validation. PCR primer pairs were designed based on the flanking sequences of the selected polymorphic sites using Primer 3 program (http://bioinfo.ut.ee/primer3-0.4.0/) and used for genomic DNA PCR analyses. Amplified fragments were analyzed by separation in agarose gels and ethidium bromide staining.

### Phylogenetic analysis and estimation of divergence time

Divergence time was first calculated based on Ks value. Ka and Ks values represent the number of non-synonymous and synonymous substitution per site, respectively. To estimate divergence time of these ten species in the Araliaceae family, 79 protein-coding sequences from each cp genome of the ten species were extracted and concatenated. Mean Ka and Ks values were calculated by pair-wise comparison of nucleotide substitution among the common cp protein-coding genes of the ten species, using the PAML program^54^. Divergence time (T) was given by T = Ks/2λ, where λ is approximately 1.0 × 10^−9^ substitutions per site per year^26^.

Second, divergence time was calculated using the Bayesian Inference (BI) method. A phylogenetic tree was generated based on BI analysis using BEAST version 1.8.1^55^ with 95% highest posterior density. The analysis was conducted with the data of 79 chloroplast protein-coding gene sequences using a strict clock approach, with Yule prior on the tree, general time reversible (GTR + I + Γ) as a substitution model and the default priors for generating a random starting tree. The maximum probability clade trees were calculated using TreeAnnotator (version 1.8.1) and root age was constrained to be 60.2 million years ago (MYA) based on previously reported divergence time between Araliaceae and Apiaceae in the order Apiales^41–44^. The final tree was visualized in FigTree v1.3.1.

The nrITS-based tree was constructed using the ML method of MEGA6.0 with 1,000 bootstrap replicates. As outgroup sequences for phylogenetic analysis, cp genome^56^ (NC_008325.1) and nrITS sequence (AY552527.1) of *Daucus carota* (carrot) belonging to the Apiaceae family were used.

## Electronic supplementary material

Supplementary Tables and Figures

Dataset S1
