# Supplementary material for: Evolution of the Araliaceae family inferred from complete chloroplast genomes and 45S nrDNAs of 10 Panax-related species
Source: Sci Rep. 2017 Jul 7;7:4917. doi: 10.1038/s41598-017-05218-y (PMC5501832; doi:10.1038/s41598-017-05218-y)
Supplement: Supplementary file 1 — Supplementary Tables and Figures [file 41598_2017_5218_MOESM1_ESM.pdf]

Evolution of the Araliaceae family inferred from complete chloroplast genomes and 45S nrDNAs of 10 *Panax*-related species

Kyunghee Kim[1†], Van Binh Nguyen[1†], Jingzhou Dong[2], Ying Wang[3], Jee Young Park[1], Sang-Choon Lee[1] and Tae-Jin Yang[1,4*]

Supplementary Tables and Figures

Supplementary Table S1. Cp genome sequence similarity among ten Araliaceae species

Species		% Identity between cp coding sequences									
		PG	PQ	PN	PJ	PV	AE	AU	ES	ESen	DM
% Identity between whole cp sequences	PG		99.6	99.5	99.6	99.2	98.6	98.8	98.4	98.5	98.5
	PQ	99.6		99.2	99.6	99.4	98.5	98.5	98.5	98.6	98.6
	PN	99.2	99.3		99.3	98.9	98.5	98.9	98.2	98.3	98.3
	PJ	99.1	99.1	99.4		99.4	98.6	98.6	98.5	98.6	98.6
	PV	99.1	99.0	99.0	98.9		98.4	98.2	98.5	98.6	98.6
	AE	97.9	98.0	97.9	97.9	97.7		99.2	98.5	98.6	98.6
	AU	97.9	97.8	97.7	97.6	97.9	99.1		98.4	98.4	98.4
	ES	97.3	97.4	97.3	97.3	97.1	97.7	97.4		99.6	99.3
	ESen	97.5	97.6	97.5	97.5	97.3	97.9	97.6	99.1		99.4
	DM	97.5	97.6	97.5	97.5	97.3	97.8	97.5	98.7	98.9	

Supplementary Table S2. Primer sets for validation of polymorphic sites in cp genomes

Primer name		Primer sequence (5' - 3')	Location	PCR Product size (bp)								
				**PG**	**PQ**	**PN**	**PJ**	**PV**	**ES**	**ESen**	**DM**	**AE**
Araliaceae 01	F	CAAGACTTCCCGATTCCCTTC	*trnC*-GCA ~ *petN*	226	226	226	226	226	520	520	538	498
	R	CCATGTGAATTATGTCCCCTATCTC										
Araliaceae 02	F	TTCCAACAGTTAGACCTTTC	*trnS*-GGA ~ *rps4*	227	227	227	227	227	309	309	309	217
	R	GATAGTAAATGGGTCGGTTTGAA										
Araliaceae 03	F	CATTCTTAGCTATTCAAATCC	*trnT*-UGU ~ *trnL*-UAA	392	401	385	386	396	384	415	351	359
	R	GTCTGAGCTTCCATCTTTACGA										
Araliaceae 04	F	CGTGTTTCTCCGTCACTTG	*cemA* ~ *petA*	195	195	195	195	195	228	228	228	218
	R	AGTTCCCTATCCGCGATTCT										
pgycf01	F	GGTATTAGTCTGGATACGGCAAA	*ycf1*	672	615	729	615	558	558	558	558	558
	R	TCGAAAAGAAGGGTCACAAGA										

Supplementary Table S3. Number of SNPs in 45S nrDNA regions of eight Araliaceae species

	Position	PG (ref)	PQ	PV	PJ	PN	AE	ES	DM
**18S** (1 – 1808 bp)	189	C	.	.	.	T	.	.	.
	232	C	.	.	.	.	.	A	T
	236	C	.	.	.	.	.	T	T
	495	G	C	C	C	C	C	C	C
	497	T	G	G	.	G	C	A	A
	499	C	G	G	G	G	G	G	G
	681	A	.	.	G	.	.	.	.
	1575	C	.	.	.	.	.	.	A
	1716	C	.	.	.	T	.	.	.
**5.8S** (2032 – 2191 bp)	2043	A	G	G	G	G	G	G	G
	2111	G	.	.	T	.	.	.	.
	2123	C	.	.	.	T	.	.	.
	2166	C	.	.	.	.	.	T	T
**26S** (2425 – 5877 bp)	2542	C	.	.	.	T	.	.	.
	2551	C	.	.	.	T	T	T	T
	2558	C	.	.	.	.	.	.	T
	2625	A	.	G	G	G	G	G	G
	2714	C	.	.	.	.	.	.	A
	2843	G	.	.	.	A	.	.	.
	2861	A	G	.	G	G	G	G	G
	2862	C	.	.	.	.	.	T	.
	2885	A	.	.	.	.	.	T	.
	2886	T	.	.	C	.	.	.	.
	2896	T	.	.	.	.	.	C	C
	2912	G	.	.	.	.	.	A	.
	2913	T	.	.	.	.	C	C	C
	2935	C	.	.	.	.	.	T	.
	2981	G	.	.	.	.	.	A	.
	2984	C	.	T	.	.	.	.	.
	2009	T	.	A	.	A	.	.	.
	3011	A	.	.	G	.	.	.	.
	3012	C	.	.	.	T	.	.	.
	3044	C	.	.	.	T	.	.	T
	3154	T	.	.	C	.	.	.	.
	3164	C	.	.	.	.	.	T	.
	3503	T	.	.	.	C	C	C	C
	3527	T	.	.	C	.	.	.	C
	3837	T	.	.	C	.	.	.	C
	3983	C	.	.	T	.	.	.	.
	4007	G	.	.	A	.	.	.	.
	4095	C	.	.	.	.	.	T	T
	4096	C	.	.	.	.	.	T	T
	4100	A	.	.	.	.	.	G	G
	4118	T	.	.	.	.	.	.	C

	4205	G	.	A	.	A	.	.	.
	4399	T	.	C	.	C	C	C	C
	4404	T	.	.	.	C	C	C	C
	4436	T	.	C	C	C	C	C	C
	4982	A	.	.	.	.	.	G	G
	4998	T	.	.	.	.	.	.	C
	5270	C	.	.	.	T	T	T	T
	5573	C	.	T	.	.	.	.	.
	5576	A	.	.	.	.	.	.	G
	5616	T	.	.	C	C	C	C	C
	5617	A	.	.	.	G	.	.	G
	5653	T	.	C	.	.	.	.	.
	5655	C	.	.	.	.	.	T	T
	5656	G	.	.	.	.	.	A	.
	5667	C	.	.	.	.	.	T	.
	5670	A	.	.	.	G	.	.	.
	5693	C	.	.	.	.	.	.	T
	5824	C	.	.	.	.	.	.	T
	5825	T	.	.	.	.	.	.	C

Supplementary Table S4. Detailed information of SNPs in nrITS (ITS1 and ITS2) regions of eight Araliaceae species

	Position	PG (ref)	PQ	PV	PJ	PN	AE	ES	DM
**ITS1** (1809 – 2031 bp)	14	T	.	.	.	C	C	C	C
	29	C	.	.	.	.	.	.	T
	35	C	.	.	.	A	.	.	.
	41	A	.	.	.	.	.	C	C
	42	A	.	.	C	.	.	.	.
	43	T	.	.	.	.	C	.	C
	57	C	.	.	.	T	.	.	.
	59	A	.	.	.	.	.	T	G
	60	G	.	.	.	.	A	.	.
	68	C	.	.	.	T	A	.	.
	69	A	.	.	.	.	.	.	G
	70	A	.	.	.	G	.	.	.
	72	C	.	.	.	.	.	T	T
	82	T	.	.	C	.	C	C	C
	85	A	.	.	.	.	G	.	G
	101	A	.	.	T	.	.	.	.
	102	C	.	.	.	.	.	T	T
	103	C	.	.	.	.	.	.	T
	104	A	.	G	.	G	G	G	G
	109	T	.	.	.	.	C	C	.
	112	G	.	.	.	.	.	T	.
	113	T	.	.	.	.	.	.	C
	115	G	.	.	.	.	T	.	.
	116	A	C	C	C	C	T	T	T
	117	T	.	.	.	C	C	C	C
	121	G	.	.	.	.	T	.	.
	122	T	.	.	.	.	.	A	.
	124	C	.	.	.	.	.	T	T
	133	A	.	.	.	.	.	T	.
	135	C	.	.	.	.	A	A	.
	163	C	.	.	.	.	.	T	.
	167	C	.	.	.	T	T	.	.
	175	C	.	.	.	.	A	A	A
	176	A	.	G	.	.	.	.	.
	185	C	.	.	.	.	.	A	A
	192	T	.	.	.	.	.	C	C
	199	G	.	.	.	.	.	T	.
	201	C	.	.	.	.	.	T	.
	205	A	.	.	.	.	G	G	G
	214	C	.	.	.	.	.	T	.
	215	T	.	.	C	.	.	.	.
	219	A	.	.	.	.	.	.	T
**ITS2** (2192 – 2424 bp)	404	C	.	T	.	.	.	.	.
	405	C	.	.	.	.	.	.	A
	406	A	.	.	G	.	C	T	C
	407	T	.	.	.	.	G	G	G
	409	A	.	.	.	.	.	.	G
	410	C	.	.	.	T	.	.	.

	414	C	T	.	.	.	.	.	.
	416	T	.	C	.	C	C	C	C
	417	G	.	A	.	.	A	A	A
	418	C	.	.	.	.	T	T	.
	423	G	.	.	.	.	.	.	A
	425	T	C	C	C	C	C	C	C
	426	G	.	.	.	.	.	A	.
	427	A	.	.	.	.	.	T	T
	428	G	.	.	.	T	.	.	.
	431	G	.	.	.	.	A	T	.
	444	A	.	.	.	.	T	C	C
	464	G	.	.	.	.	A	.	.
	465	C	.	.	.	.	.	T	.
	482	C	.	.	.	.	T	T	T
	495	A	.	.	.	.	.	G	.
	496	T	.	.	.	.	C	C	C
	522	A	.	.	.	T	.	.	.
	538	A	.	.	.	.	.	C	C
	547	G	.	.	.	.	.	T	.
	551	A	.	.	.	.	.	.	C
	559	C	.	.	.	.	.	T	.
	560	C	.	.	.	.	.	.	T
	562	G	.	.	.	.	.	.	A
	564	A	.	.	.	.	.	.	C
	567	A	.	.	G	.	G	.	.
	568	G	.	.	.	.	.	.	A
	572	T	.	.	.	.	.	A	.
	573	C	.	.	.	.	.	T	.
	574	A	.	.	.	.	.	G	G
	575	T	.	.	A	.	.	.	.
	586	C	.	.	.	.	.	T	T
	589	C	.	.	.	T	.	.	.
	592	C	.	.	A	.	.	.	.
	595	C	.	.	A	.	.	.	.
	598	C	.	T	.	.	.	.	.
	600	T	.	C	C	C	C	A	C
	603	G	.	.	.	.	.	A	A
	610	C	.	.	.	.	.	.	A

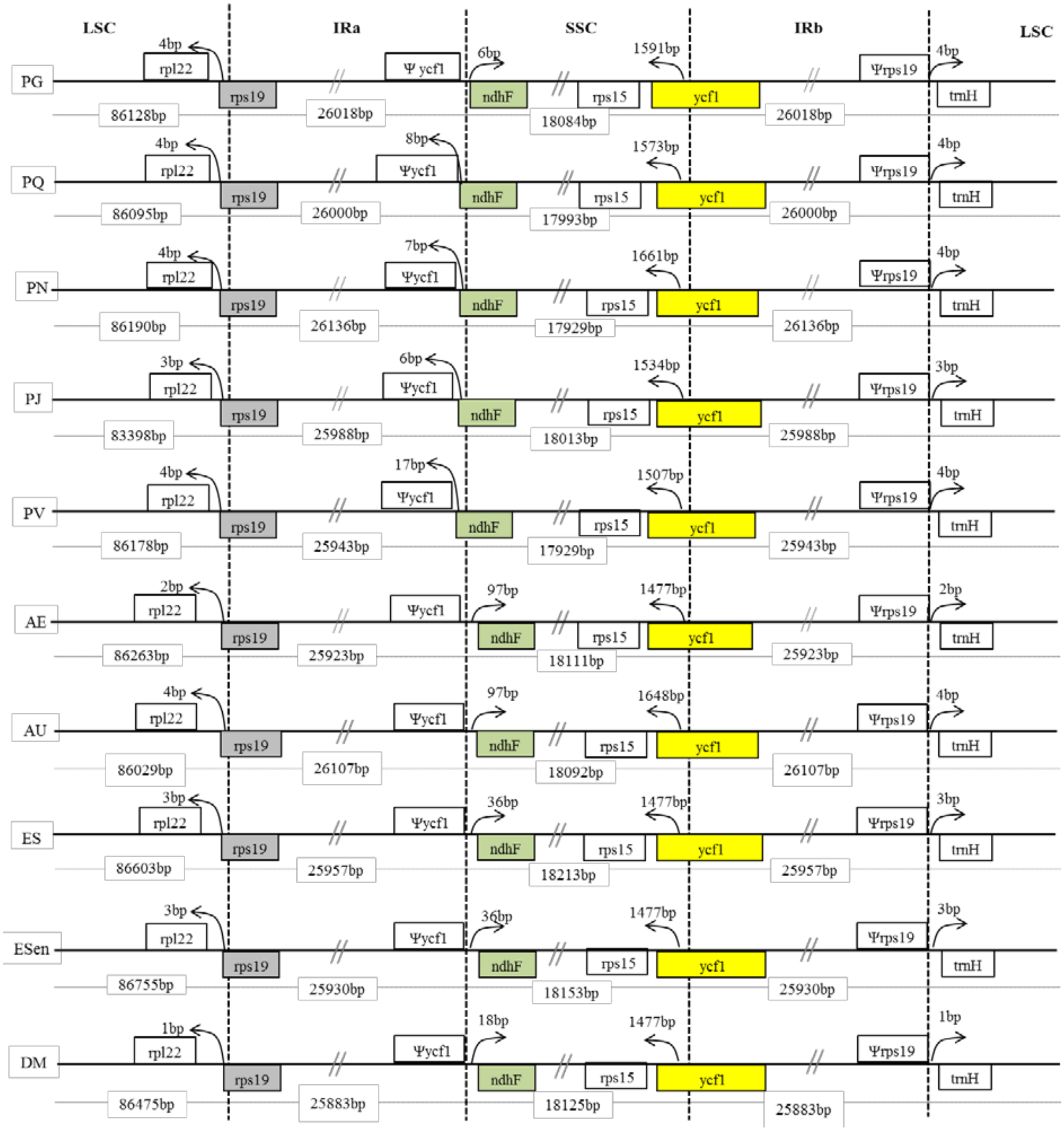

Supplementary Fig. S1. Comparison of border regions among cp genomes in 10 Araliaceae species.

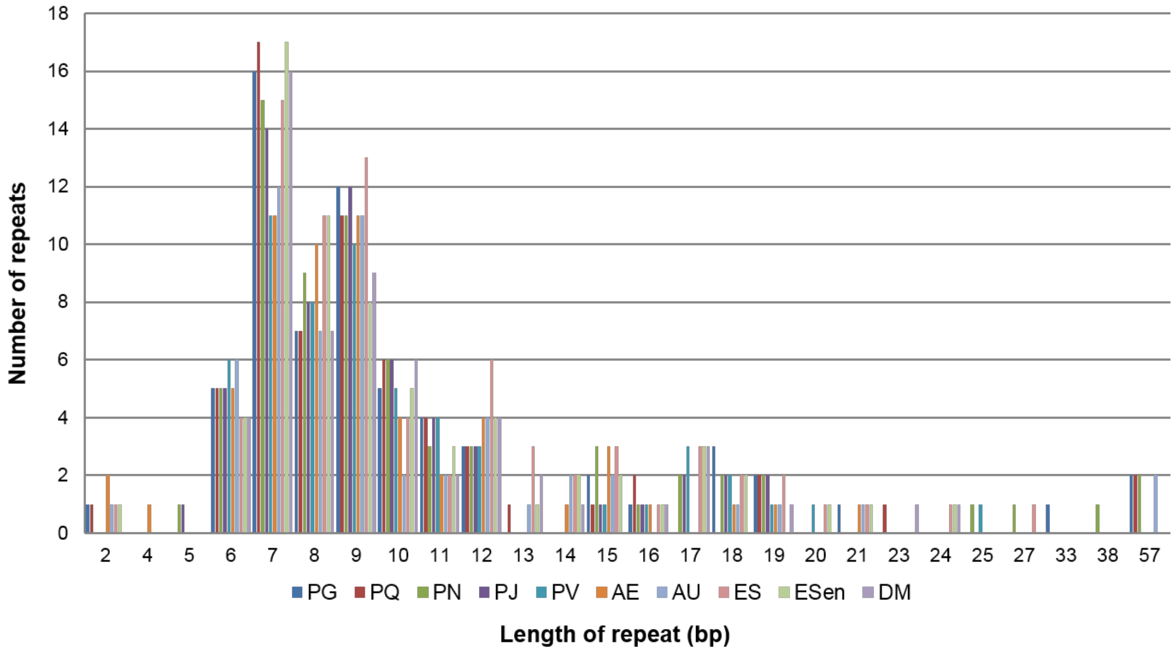

Supplementary Fig. S2. Distribution of tandem repeats (TRs) in cp genomes of ten Araliaceae species.

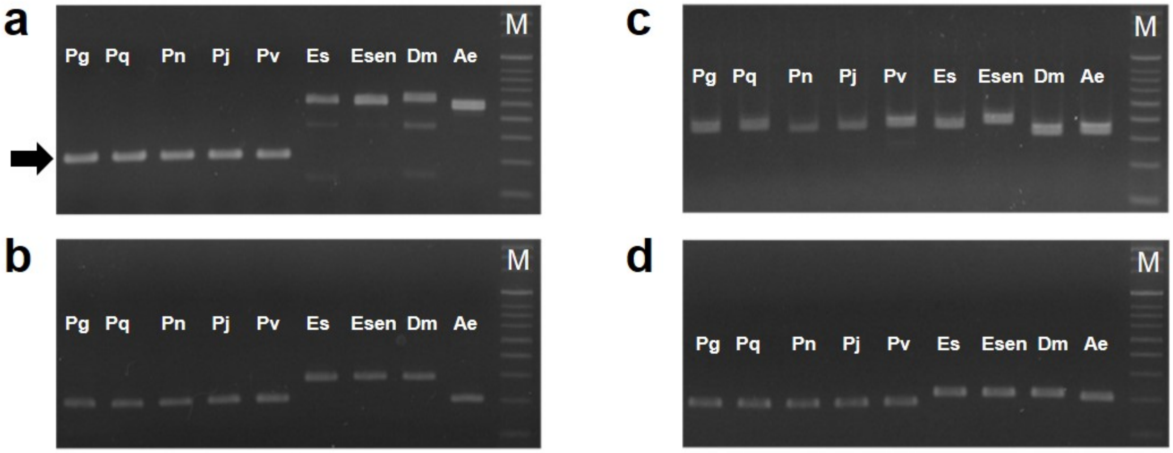

Supplementary Fig. S3. Validation of polymorphic sites found in cp genomes of Araliaceae species. (a) INDEL at *trnC*-GCA ~ *petN*. (b) TR CNV at *trnS*-GGA ~ *rps4*. (c) TR CNV at *trnT*-UGU ~ *trnL*-UAA. (d) TR CNV at *cemA* ~ *petA*. Black arrow indicates the specific bands for *Panax* genus. Abbreviated species names (Table 1) are shown above the lanes. M indicate 100-bp DNA ladder

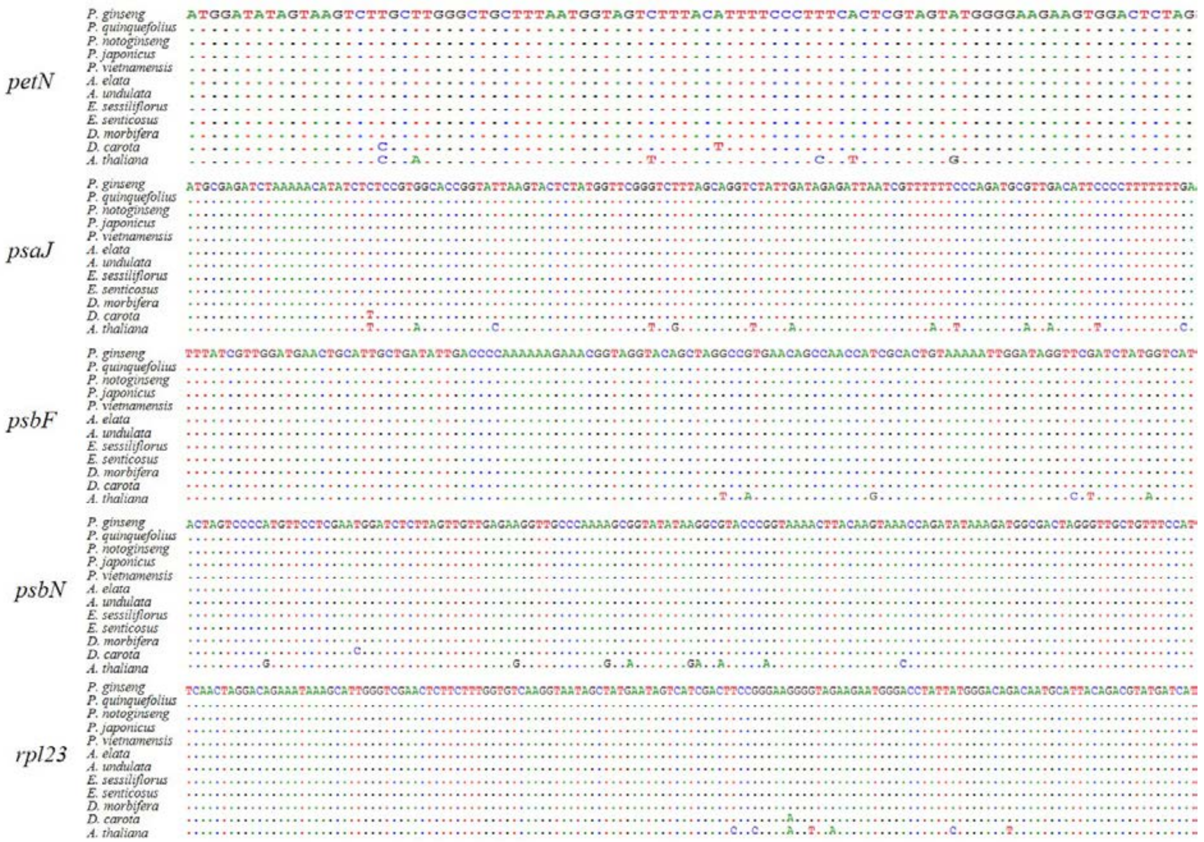

Supplementary Fig. S4. Sequence comparison of five cp conserved genes in the Araliaceae family.
